# Social Media and High-Risk Eating Behaviors in Adults: A Cross-Sectional Study

**DOI:** 10.3390/healthcare14050666

**Published:** 2026-03-06

**Authors:** Eman Khalid Alqadheeb, Peter M. B. Cahusac, Narmeen Shaikh, Noara Alhusseini

**Affiliations:** 1 Department of Biostatistics, Epidemiology and Public Health, College of Medicine, Alfaisal University, Riyadh 11533, Saudi Arabia; ealqadheeb@alfaisal.edu (E.K.A.); nalhusseini@alfaisal.edu (N.A.); 2 Department of Pharmacology and Biostatistics, College of Medicine, Alfaisal University, Riyadh 11533, Saudi Arabia; pcahusac@alfaisal.edu

**Keywords:** high-risk disordered eating, disordered eating behaviors, social media, Saudi Arabia, BMI

## Abstract

**Introduction**: Eating disorders and disordered eating behaviours are crucial mental health concerns, yet evidence linking social media use to eating-related outcomes in adult populations, particularly in Saudi Arabia, remains limited and primarily focused on female or student samples. This study examined the prevalence of disordered eating risk among Saudi adults and its association with social media engagement, body mass index (BMI), and sociodemographic factors. **Method**: A cross-sectional survey was conducted among adults residing in Saudi Arabia. Disordered eating risk was assessed using the Eating Attitudes Test (EAT-26), and social media engagement was measured as frequency of use across multiple daily routines using the Social Media Engagement Questionnaire (SMEQ). BMI and sociodemographic variables were self-reported. Descriptive statistics, bivariate analyses, and multivariable linear regression were performed to examine associations between study variables. **Results**: Approximately 43% of respondents were at high risk of disordered eating behaviors. Social media engagement was prevalent; however, its association with disordered eating risk was weak and inverse (Spearman’s Rho = −0.1243, *p* < 0.01). BMI showed domain-specific associations across eating-related domains, while most sociodemographic factors were not strongly associated with disordered eating outcomes. **Conclusions**: Disordered eating behaviors were present among adults in Saudi Arabia and extend beyond traditionally studied high-risk groups. The weak inverse association indicates that frequency of social media use alone may not be a reliable indicator of eating-related risk in adults and likely does not capture content- and comparison-specific mechanisms. Findings highlight the need for broader screening and prevention efforts, as well as for longitudinal research using content- and behavior-specific measures of social media exposure to clarify mechanisms in adult populations.

## 1. Introduction

Disordered eating behaviors (DEBs) constitute a significant and growing public health concern. DEBs represent a broad spectrum of maladaptive eating-related practices in the general population (e.g., restrictive dieting, fasting, binge eating, purging, and misuse of weight-control substances) that may occur without a formal eating disorder diagnosis [1]. Clinically diagnosed eating disorders (EDs), such as anorexia nervosa, bulimia nervosa, and binge eating disorder, represent the more severe end of this spectrum [2]. From a clinical and epidemiological perspective, DEBs are particularly important because they are far more prevalent than diagnosed EDs, frequently remain undetected due to their subclinical nature, and are linked to significant psychological distress and functional impairment [3,4,5]. Importantly, these behaviors may precede or progress to diagnosable eating disorders [5], underscoring their relevance as a key target for early identification and prevention.

A growing body of evidence suggests that DEBs and EDs are not uncommon in Western Asia and Arab populations. A comprehensive literature review reported prevalence estimates ranging from approximately 2% to over 50%, reflecting both true heterogeneity and methodological differences across studies [6]. Similarly, a meta-analysis of Western Asia estimated a pooled prevalence of disordered eating behaviors of 22% [7]. However, most existing studies in the region have focused on adolescents, university students, or single-gender samples, limiting generalizability to the adult population and obscuring potential sex- and age-related patterns.

Disordered eating is multifactorial, reflecting interactions among psychological, biological, and sociocultural factors [8,9]. In recent years, digital environments, particularly social media platforms, have emerged as influential sociocultural exposures linked to body dissatisfaction and eating-related psychopathology [10,11,12]. Several theoretical frameworks help explain how this influence may operate. The Tripartite Influence Model [13,14] proposes that sociocultural pressures from peers, family, and media promote body dissatisfaction and disordered eating through two mediating pathways: internalization of appearance ideals and appearance-based social comparison. Within this framework, social media functions as a potent media channel that amplifies exposure to idealized body images and facilitates upward social comparison [15]. This mechanism is rooted in Social Comparison Theory [16], which posits that individuals evaluate themselves by comparing with others on salient dimensions such as appearance; when such comparisons are upward, they tend to increase body dissatisfaction and eating-related distress [17]. Critically, both frameworks predict that the link between social media and disordered eating operates through specific content exposure and comparison processes rather than through general use frequency. This distinction is supported empirically: content-specific and comparison-specific measures of social media use have shown more consistent associations with disordered eating than overall time spent online [10,11]. Moreover, Uses and Gratifications Theory [18] suggests that individuals actively select media to satisfy particular needs. For example, individuals may use social media to facilitate social support and beneficial mental health-related interactions [19] rather than appearance-focused browsing, which could potentially weaken the expected association between general engagement frequency and disordered eating. International evidence on social media use and disordered eating has yielded mixed findings: several cross-sectional studies and meta-analyses report positive associations [20,21,22], whereas others report weak or null associations [23]. These inconsistencies may reflect differences in measurement approach (frequency-based vs. content-specific), study populations (adolescents vs. adults), platform-specific behaviors, and cultural contexts [24], highlighting the need for population-specific research guided by clear theoretical expectations.

In Saudi Arabia, social media use is widespread, with a large proportion of the population engaging daily across multiple platforms [25]. Several Saudi studies have reported associations between social media use and eating-related outcomes, including high-risk disordered eating behaviors and body image concerns [26,27,28,29,30,31]. For example, in a study with 1884 participants, engagement with influencer content emerged as a key predictor of body image issues and eating-disorder symptoms [27]. However, existing research remains concentrated within specific subgroups (e.g., females, adolescents, and university students), a pattern that mirrors broader Arab world-focused reviews [32], and limits generalizability to adult and mixed-gender community samples. Furthermore, BMI may shape the relationship between social media engagement and disordered eating symptoms, but has not been consistently considered in Saudi studies examining this association. BMI may act as a confounder, given established links between higher BMI and disordered eating symptoms [33,34] and because weight status may also relate to patterns of social media use [35]. BMI may also function as a moderator, whereby appearance-focused content and social comparison could have stronger associations with eating-related symptoms among individuals with higher BMI due to heightened weight stigma or body dissatisfaction [24]. BMI could also plausibly lie on a mediating pathway (e.g., social media-related body dissatisfaction influencing eating behaviors and weight change over time) [24]; however, such pathways require longitudinal data for evaluation. Thus, Saudi studies would benefit from examining social media engagement in relation to disordered eating behaviors while considering BMI as a relevant correlate and potential source of heterogeneity.

To address these gaps, the present study aimed to (1) estimate the prevalence of elevated risk disordered eating behaviors (DEBs) among adults in Saudi Arabia using the Eating Attitudes Test-26 (EAT-26), a validated screening instrument that identifies individuals at elevated risk rather than establishing clinical diagnoses, and (2) examine the association between social media engagement and disordered eating risk. As secondary objectives, we assessed the relationships between BMI, social media use, and disordered eating risk, and explored associations with key sociodemographic characteristics. By focusing on a mixed-gender adult population, this study seeks to contribute context-specific evidence to an area characterized by inconsistent findings and limited regional data, with implications for prevention, screening, and public health interventions in digitally connected societies.

### Research Questions

1. What is the prevalence of high-risk disordered eating behaviors (EAT-26 ≥ 20) among adults residing in Saudi Arabia?
2. Is frequency-based social media engagement (SMEQ) associated with EAT-26 outcomes (total and symptom domains)?
3. In multivariable models, are SMEQ, BMI, and sociodemographic characteristics (e.g., age group and gender) independently associated with EAT-26 outcomes?

## 2. Methods

### 2.1. Study Design, Study Population, and Eligibility

A cross-sectional study was conducted to examine the association between high-risk disordered eating behaviors and social media use among adults residing in Saudi Arabia. Data were collected over eight months, from January to August 2023, using a self-administered online questionnaire available in both Arabic and English. Eligible participants were adults aged 18 years or older who were currently residing in Saudi Arabia and who provided informed consent before participation. Individuals younger than 18 years, those not residing in Saudi Arabia, or those who declined participation were excluded from the study.

### 2.2. Sample Size and Data Collection Procedure

The minimum required sample size was calculated based on the estimated population of Saudi Arabia (36,408,820) [25] and a 95% confidence level, yielding a minimum sample size of 385 participants. This calculation was performed using the Raosoft online sample size calculator. To improve generalizability and statistical power, a larger sample was targeted. A total of 641 participants completed the survey, exceeding the minimum required sample size by approximately 66%.

A non-probability sampling approach was used, combining convenience sampling and snowball sampling. The survey link was disseminated via social media platforms, including Twitter (X), WhatsApp, LinkedIn, and Facebook. Participants were also encouraged to share the survey within their social networks. Because recruitment occurred primarily through social media and participants self-selected into the study, individuals with higher social media use may have been more likely to participate, introducing potential selection bias.

Data were collected using Google Forms, which hosted the anonymous questionnaire. The survey was distributed through direct links and Quick Response (QR) codes embedded in social media posts and messages. Participation was voluntary, and respondents could discontinue the survey at any time without penalty. No personally identifiable information was collected.

### 2.3. Measurement Instruments

We employed two validated questionnaires, the EAT-26 and the SMEQ, along with key sociodemographic information, and presented them in both Arabic and English [36,37].

#### 2.3.1. Sociodemographic and Anthropometric Variables

Participants self-reported sociodemographic information, including age, gender, nationality, marital status, educational level, employment status, monthly income, and province of residence. Self-reported height and weight were used to calculate body mass index (BMI), which was categorized according to World Health Organization classification criteria. Because height and weight were self-reported, BMI estimates may be subject to reporting error (e.g., under-reporting of weight), which could attenuate associations involving BMI.

#### 2.3.2. Disordered Eating Risk

Risk of disordered eating behaviors was assessed using the Eating Attitudes Test-26 (EAT-26), a widely used screening instrument for identifying individuals at risk of eating disorders [37]. The questionnaire consists of 26 items scored on a six-point Likert scale. Responses for items 1–25 are scored on a 4-point scale: “Always” (3 points), “Usually” (2 points), “Often” (1 point), and “Sometimes,” “Rarely,” and “Never” (0 points each). Item 26 is reverse-scored, and a final score is calculated by summing items 1–26 [38]. Total scores range from 0 to 78, with a score of ≥20 indicating a high risk of disordered eating behaviors. Subscale scores were calculated by summing item responses as follows: Dieting (items 1, 6, 7, 10, 11, 12, 14, 16, 17, 22, 23, 24, 26), Bulimia and Food Preoccupation (items 3, 4, 9, 18, 21, 25), and Oral Control (items 2, 5, 8, 13, 15, 19, 20). The Arabic version of the EAT-26 has demonstrated good validity and reliability in previous studies [39]. In the present study, internal consistency was excellent, with a Cronbach’s alpha of 0.94.

#### 2.3.3. Social Media Use

Social media engagement was assessed using the Social Media Engagement Questionnaire (SMEQ). This validated five-item instrument measures the frequency of social media use during daily routines (e.g., before bedtime, during meals, and upon waking), reflecting habitual use patterns. Importantly, the SMEQ does not differentiate between active and passive engagement, time spent, or content type (e.g., appearance- or fitness-related material). As such, the measure represents general frequency-based engagement rather than qualitative exposure to specific social media content.

Each item is scored on a seven-point scale ranging from 0 (never) to 7 (every day). A composite SMEQ score was calculated by summing item responses, with higher scores indicating greater engagement. The Arabic version of the SMEQ has previously demonstrated acceptable validity and reliability for use in Arabic-speaking populations [40].

### 2.4. Statistical Analysis

Statistical analyses were conducted using Jamovi (version 2.4) and Microsoft Excel. Descriptive statistics were used to summarize sociodemographic characteristics and questionnaire scores. Categorical variables were reported as frequencies and percentages, while continuous variables were summarized using means and standard deviations.

Normality of continuous variables was assessed, and given non-normal distributions, Spearman’s rank correlation coefficient (ρ) was used to examine associations between EAT-26 scores, SMEQ scores, BMI, and sociodemographic variables. Correlation strength was interpreted according to standard guidelines.

Multiple linear regression analyses were conducted to assess independent associations between participant characteristics and eating-disorder symptom scores. Four models were estimated using (1) EAT-26 total score and (2–4) each EAT-26 subscale score (Dieting; Bulimia and Food Preoccupation; Oral Control) as dependent variables. Predictor variables included gender, BMI, composite social media score, and age group. BMI was included a priori as a potential confounder because it is plausibly associated with both disordered eating symptom scores and patterns of social media engagement. Mediation and moderation were not evaluated because temporal ordering cannot be established in cross-sectional data, and interaction testing was beyond the scope of the current analyses.

Additional sociodemographic variables (nationality, marital status, employment status, monthly income, and education level) were examined using forward and backward stepwise selection procedures but were not retained in the final models because they did not improve model fit. Model assumptions were assessed before interpretation. Multicollinearity was evaluated using variance inflation factors (VIFs), which did not exceed 1.14. Q–Q plots and residual plots indicated acceptable model fit, and influential observations were assessed using Cook’s distance (maximum 0.08). Although formal normality tests were statistically significant, these tests can be overly sensitive in large samples; given *n* = 634 and the Central Limit Theorem, regression coefficient estimates were considered robust. Model fit was summarized using R^2^ and adjusted R^2^.

Statistical significance was defined as a two-sided *p*-value < 0.05.

### 2.5. Ethical Considerations

Ethical approval was obtained from the Institutional Review Board of Alfaisal University (IRB Log Number: IRB-20187; approval date: 12 December 2022). Informed consent was obtained electronically from all participants before survey completion. Data were anonymized and stored securely in encrypted electronic files accessible only to the research team.

## 3. Results

### 3.1. Participant Characteristics

A total of 641 participants completed the web-based questionnaire and were included in the analysis (Table 1). Of these, 54.1% were female (*n* = 347) and 45.9% were male (*n* = 294). The majority of participants were aged 18–30 years (78.9%), were Saudi nationals (93.8%), and were single (79.7%). Over half of respondents (51.5%) held a bachelor’s degree, and 62.2% were unemployed, reflecting the high proportion of students in the sample.

Regarding anthropometric characteristics, 39.0% of participants had a normal BMI (18.5–24.9 kg/m^2^), 23.1% were overweight, and 20.1% were obese (BMI ≥ 30 kg/m^2^). TikTok was the most frequently reported social media platform (27.6%), followed by Snapchat (19.3%) and Instagram (18.4%)

### 3.2. Prevalence of High-Risk Disordered Eating

**Figure 1:**
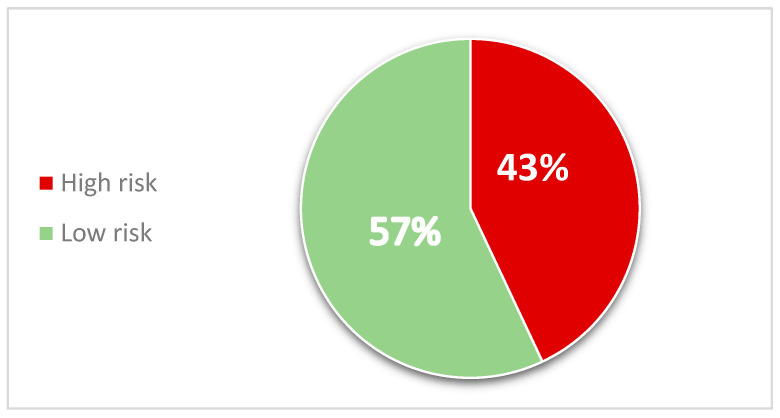
Risk levels of eating disorders among participants (*n* = 641), based on EAT-26 cut-off. High-risk disordered eating: EAT-26 total score ≥ 20. Low-risk: EAT-26 total score < 20.

Based on the EAT-26 cutoff score (≥20), approximately 43% of participants were classified as being at high risk for disordered eating behaviors (Figure 1).

Item-level responses revealed notable levels of weight-related anxiety, food preoccupation, and dieting behaviors. The most highly endorsed EAT-26 items (collapsed as “Often–Always”) are summarized in Table 2, while the complete item-level response distribution for all 26 items is provided in Supplementary Table S1. Overall, the highest endorsements clustered within the Dieting domain, with additional elevated endorsement of selected Oral Control and Bulimia/Food Preoccupation items. Approximately 59.6% of participants reported that they are often/usually/always terrified of gaining weight, and 54.9% reported often/usually/always having a desire to be thinner (Table 2). In contrast, compensatory behaviors, such as vomiting, were less commonly reported: 66.3% reported never vomiting after eating (Table S1).

### 3.3. Social Media Engagement Patterns

Social media engagement varied by time of day. The highest frequency of use was reported 15 min before bedtime, with 52.6% of participants reporting daily use, followed by 34.9% reporting daily use within 15 min of waking up. Mean SMEQ scores ranged from 3.4 ± 2.7 times per week during lunch to 5.0 ± 2.4 times per week before bedtime, indicating widespread habitual use of social media across daily routines (Table 3). Detailed routine-specific frequency distributions are provided in Supplementary Table S2.

### 3.4. Bivariate Associations: Eating-Disorder Risk, Social Media Engagement, BMI, and Demographics

Spearman’s rank correlation analysis demonstrated a statistically significant but weak negative correlation between EAT-26 total score and SMEQ composite score (ρ = −0.1243, *p* = 0.0016). This indicates that higher levels of disordered eating risk were associated with slightly lower social media engagement; however, the magnitude of this association was small.

BMI was positively but weakly correlated with the EAT-26 total score (ρ = 0.1566, *p* < 0.05), indicating a higher risk of disordered eating among individuals with higher BMI. In contrast, no significant correlation was observed between BMI and SMEQ composite score (ρ = −0.0224, *p* = 0.5732).

The associations with different sociodemographic characteristics varied with outcome and were as follows:

- BMI showed moderate positive correlations with several sociodemographic variables, including age group, educational level, gender, employment status, and monthly income (all *p* < 0.0001). No significant association was observed between BMI and nationality.
- EAT-26 total score was not significantly correlated with any sociodemographic variable, including age, gender, education, nationality, employment status, or income.
- SMEQ composite score demonstrated weak negative correlations with age group, educational level, and monthly income, indicating slightly lower social media engagement among older, more educated, and higher-income participants. No significant associations were observed between SMEQ score and gender, nationality, or employment status.

### 3.5. Multivariable Regression Analyses

In the EAT-26 total score model, higher BMI was independently associated with higher total EAT-26 scores (B = 0.340, *p* = 0.003). In contrast, higher composite social media scores (B = −0.267, *p* < 0.001) and older age group (B = −2.240, *p* = 0.020) were associated with lower total scores; gender was not statistically significant. Overall model fit was modest (R^2^ = 0.045; adjusted R^2^ = 0.039; *n* = 634). All results are shown in Table 4.

For the Dieting subscale, BMI was positively associated (B = 0.40, *p* < 0.001) and composite social media score was inversely associated (B = −0.14, *p* < 0.001), while gender and age group were not significant predictors (R^2^ = 0.080; adjusted R^2^ = 0.075).

For the Bulimia and Food Preoccupation subscale, BMI was positively associated (B = 0.11, *p* < 0.001), composite social media score was inversely associated (B = −0.06, *p* < 0.001), and older age group was associated with lower scores (B = −0.96, *p* < 0.001); gender was not significant (R^2^ = 0.045; adjusted R^2^ = 0.039).

For the Oral Control subscale, BMI (B = −0.17, *p* < 0.001), composite social media score (B = −0.07, *p* < 0.001), and older age group (B = −0.63, *p* = 0.031) were inversely associated with oral control scores; gender was not significant (R^2^ = 0.078; adjusted R^2^ = 0.072).

## 4. Discussion

In this study of adults residing in Saudi Arabia, a substantial proportion of participants screened positive for high-risk disordered eating behaviors, reinforcing growing evidence that subclinical eating-related psychopathology represents a significant public health concern in the region [6,7]. By examining a mixed-gender adult sample rather than focusing primarily on university students or women, as has been common in prior Saudi studies, the present study extends existing knowledge. It suggests that vulnerability to disordered eating behaviors may be more widely distributed across the adult population than previously assumed. At the item level, the most frequently endorsed behaviors clustered within the Dieting subdomain, particularly weight-related anxiety, desire for thinness, and preoccupation with body fat, while compensatory behaviors such as vomiting were less commonly reported. This pattern is consistent with evidence that cognitive and attitudinal symptoms of disordered eating (e.g., dietary restraint and weight preoccupation) are more prevalent than purging behaviors in non-clinical populations [41], and it suggests that screening efforts in this context should attend to the full spectrum of eating-related attitudes rather than focusing narrowly on behavioral indicators. In multivariable regression models, BMI, age group, and composite social media score were each independently associated with EAT-26 symptom scores; however, the overall explained variance was modest, reinforcing the multifactorial nature of disordered eating and indicating that unmeasured psychological, cultural, and contextual factors likely account for substantial additional variance.

Despite widespread social media engagement, higher composite social media scores were consistently and inversely associated with EAT-26 total scores and all three symptom domains (Dieting, Bulimia/Food Preoccupation, and Oral Control), although effect sizes were modest. This finding contrasts with much of the international literature, which reports positive associations between social media use and disordered eating attitudes or behaviors [20,21,22]. However, several important considerations are necessary before interpreting this pattern. First, given the cross-sectional design, the direction of the relationship cannot be determined, and these data do not support causal or functional interpretations. Reverse causality is also possible: individuals experiencing greater eating-related distress may disengage from social media or report lower use, although this pathway has not been directly tested. Second, the SMEQ measures general frequency of engagement across daily routines and does not differentiate between content types (e.g., appearance-focused vs. informational material), modes of use (e.g., passive browsing vs. active communication), or platform-specific behaviors. Because the Tripartite Influence Model [13,14] and Social Comparison Theory [16,17] predict that the pathway from media exposure to disordered eating operates primarily through internalization of appearance ideals and upward appearance comparison [15], a frequency-based measure that aggregates across heterogeneous activities may not capture the specific mechanisms most directly implicated by theory. This has also been shown in prior research [11]. Third, we hypothesize that adult populations may differ from the adolescent and student samples that dominate existing research in their motivations for social media use; a Uses and Gratifications perspective [18] would suggest that adults engage with social media for a wider range of purposes, many of which are unrelated to appearance comparison. These purposes may include communication, information seeking, and social support, which may attenuate the expected positive association between engagement frequency and disordered eating in this population, as observed in younger or more appearance-oriented user groups [19,42]. This does not imply that social media is benign or beneficial for adults; rather, that frequency of use alone is an insufficient indicator of the exposure pathways that theory identifies as most relevant to disordered eating. Taken together, these considerations suggest that the inverse association observed here most likely reflects measurement limitations and population differences rather than a substantive protective effect, and they highlight the importance of examining qualitative dimensions of social media use, particularly content exposure and social comparison behavior, in future longitudinal research with adult populations.

The bivariate and multivariable association between BMI and EAT-26 total score was positive, suggesting that confounding by the included covariates does not substantially account for this relationship. However, residual confounding by unmeasured variables (e.g., body image dissatisfaction, weight stigma) cannot be excluded. BMI also showed a differentiated pattern across EAT-26 subdomains in the multivariable models. Higher BMI was independently associated with higher Dieting and Bulimia/Food Preoccupation scores, consistent with evidence linking higher weight status to greater dieting pressure, weight concern, and compensatory eating-related behaviors [43,44,45]. In contrast, BMI was inversely associated with Oral Control scores, suggesting that this subscale may capture a more restrictive symptom profile characteristic of lower-BMI presentations. This divergence reinforces the value of interpreting EAT-26 subscales separately rather than assuming uniform relationships across symptom domains.

Gender was not significantly associated with EAT-26 total or subscale scores in any model. In contrast, the age group showed an inverse association with EAT-26 total score and with Bulimia/Food Preoccupation and Oral Control symptom domains. The absence of a gender difference is consistent with at least one prior Saudi study [31], although other Saudi research has reported a higher risk among females [28]. Internationally, sociodemographic gradients may be more pronounced in certain settings, with higher odds of disordered eating reported among younger individuals, women, those with higher BMI, and unmarried individuals, as well as evidence linking socioeconomic disadvantage to disordered eating among adult men in specific populations [46,47]. While the absence of strong sociodemographic patterning in our adult sample is itself noteworthy, the age findings warrant closer examination because the association was not uniform across EAT-26 subdomains. Age was inversely associated with Bulimia/Food Preoccupation and Oral Control scores but not with Dieting. Dieting items capture cognitive preoccupations with weight (fear of weight gain, desire for thinness, calorie awareness) that reflect culturally pervasive weight-management norms, which are likely to persist into adulthood, particularly in contexts of rising obesity prevalence and widespread diet-related messaging [48]. In contrast, the Bulimia/Food Preoccupation subscale captures more behaviorally dysregulated symptoms such as binge eating, which are consistent with evidence that impulsivity and emotional dysregulation are more pronounced in younger adults and decline with age [49,50]. The Oral Control subscale includes socially embedded items (e.g., feeling pressured to eat, being perceived as too thin) that may be more salient for younger adults navigating peer-dense environments in which eating is subject to greater interpersonal scrutiny. Together, these patterns suggest that cognitive weight concerns may remain relatively stable across adulthood, while behavioral and socially driven eating symptoms attenuate with age.

Regarding social media engagement and sociodemographic patterns, SMEQ scores were modestly lower among older, more educated, and higher-income participants. Population studies indicate that older adults engage with social media less frequently and more selectively than younger users, often due to lower digital literacy, usability, and privacy concerns, as well as a preference for maintaining a small circle of close ties rather than broad online networks [51]. We speculate that higher-SES adults, particularly those who are older and more educated, may also use social media more instrumentally, relying more on offline resources and professional networks, which could contribute to modestly lower overall engagement intensity. This clustering is important because it suggests that SMEQ frequency may reflect distinct underlying exposure profiles across age and SES groups, thereby limiting the interpretability of a single pooled association between SMEQ frequency and disordered eating.

From a public health perspective, these findings highlight the importance of addressing disordered eating behaviors as a population-level concern in Saudi Arabia. The high proportion of participants screening above the EAT-26 clinical threshold, combined with the subclinical nature of most endorsed behaviors, predominantly cognitive and attitudinal symptoms such as weight anxiety and desire for thinness rather than overt purging, suggests that many affected individuals are unlikely to be identified through routine clinical pathways that rely on recognition of behavioral indicators. This supports the case for integrating brief, validated screening tools, such as the EAT-26, into preventive health visits, university health screenings, and occupational health checks, where at-risk individuals might otherwise remain undetected. School-based strategies introduced as early as secondary education may further support early detection and prevention [52]. Regarding digital health interventions, the finding that general social media frequency was not positively associated with disordered eating risk, and that the theoretical frameworks reviewed suggest content exposure and social comparison processes as the more relevant mechanisms, indicates that programs targeting media literacy, critical appraisal of appearance-related content, and body image resilience may be more justified than approaches focused primarily on reducing screen time [11,53]. However, the cross-sectional design and modest explained variance of the regression models mean that these recommendations should be considered preliminary. The unexplained variance points to broader psychosocial determinants (such as body image dissatisfaction, weight stigma, and mental health comorbidities) that were not captured in the present analyses and should be incorporated into future studies. Longitudinal designs are needed to clarify the temporal ordering and potential bidirectionality of associations between social media engagement and disordered eating, with greater attention to content-specific and comparison-specific measures. In the Saudi context, prioritizing community-based samples with adequate representation of men and older adults, and where feasible, incorporating objective digital-use measures (e.g., device-generated logs, screen-time tracking), will be critical to improving the relevance and precision of findings.

### Strengths and Limitations

This study has several important strengths. It addresses a clear gap in the Saudi literature by examining disordered eating behaviors and social media engagement in a community-based adult sample that includes both men and women. The large sample size enhances statistical power and supports more stable estimates across analyses. The use of validated Arabic versions of established instruments (EAT-26 and the Social Media Engagement Questionnaire) strengthens measurement reliability and cultural appropriateness. In addition, including body mass index (BMI) alongside social media engagement and sociodemographic variables provides a more comprehensive analytical framework than many prior studies, enabling the examination of complex, potentially confounded relationships. Moreover, analyzing EAT-26 subscale scores alongside the total score revealed domain-specific associations, particularly for BMI and age, that would have been obscured by reliance on a single composite measure, reinforcing the value of disaggregated symptom assessment. Finally, assessing social media engagement across multiple daily routines offers a broader representation of habitual digital behavior than single-item or time-only measures.

Several limitations should also be acknowledged. The cross-sectional design precludes causal inference and limits conclusions regarding the directionality of associations between social media engagement and disordered eating behaviors. In particular, the inverse social media association cannot be interpreted as evidence of a protective or functional role of social media. Relatedly, the cross-sectional design prevents clarifying the role of BMI as a mediator in the relationship between social media engagement and disordered eating symptoms. Although BMI was adjusted for as a covariate to reduce confounding, we did not assess effect modification (e.g., social media × BMI interaction), and associations may differ across BMI strata. All data were self-reported, which may introduce recall bias or social desirability bias, particularly for sensitive behaviors related to eating, weight, and digital use. If participants underreport socially undesirable behaviors (e.g., vomiting, binge eating, or high levels of social media use), true symptom levels and engagement may be underestimated, which could attenuate observed associations and reduce effect sizes. Conversely, misclassification of exposure (social media engagement) or outcomes (EAT-26 symptoms) due to inaccurate recall could introduce non-differential measurement error that typically biases associations toward the null, although if reporting accuracy varies systematically by symptom severity, the direction of bias is uncertain. Self-reported height and weight may introduce measurement error into BMI estimates, potentially weakening BMI-related estimates. Although social media engagement was assessed using a structured questionnaire, the measure captured frequency of use rather than the type of activity (e.g., passive browsing vs. active posting) or exposure to specific content types (e.g., appearance- or fitness-related material), which may be more proximately related to eating-related psychopathology. Additionally, the use of composite SMEQ and EAT-26 scores improves reliability and parsimony but reduces interpretability of the underlying mechanisms, as it does not distinguish which specific routines, platforms, or content types drive the observed associations. The use of non-probability sampling limits generalizability to the broader Saudi adult population, and the sample included a high proportion of younger adults, which may have influenced the observed patterns. In addition, recruitment through social media platforms may have introduced self-selection bias, as individuals who use social media more frequently could have been more likely to encounter the survey link and participate. This selection process could bias the estimated association in either direction and may also inflate the sample’s overall social media engagement estimates. Consequently, prevalence estimates and effect sizes should be interpreted with caution. Additionally, other potentially relevant psychosocial factors, such as body image dissatisfaction, internalized weight stigma, and mental health comorbidities, were not measured and may partially explain observed associations.

## 5. Conclusions

This study provides novel evidence on disordered eating behaviors and social media engagement among adults in Saudi Arabia, a population that has been underrepresented in existing research. A substantial proportion of participants screened positive for elevated risk of disordered eating. In adjusted analyses, higher frequency-based social media engagement was modestly and inversely associated with EAT-26 total and subscale scores, while BMI and age showed domain-specific associations with eating-related symptom patterns. The findings emphasize the need to view disordered eating behaviors as a population-level concern that warrants broader screening and prevention efforts in Saudi Arabia. They also generate hypotheses that approaches focused on screen time alone may be insufficient; future research should evaluate whether content exposure, engagement patterns, media literacy, and psychosocial factors better explain eating-related risk. Overall, the results can inform longitudinal and mechanistic studies needed to guide culturally appropriate public health strategies in digitally connected societies.

## Figures and Tables

**Table 1:** Sociodemographic Characteristics of the Respondents (*n* = 641) *.

		N	%
**Gender**	Male	294	45.9
	Female	347	54.1
**Age**	18–30	506	78.9
	31–40	97	15.1
	41–50	22	3.4
	51–60	13	2.0
	>60	3	0.5
**Nationality**	Saudi	601	93.8
	Non-Saudi	40	6.2
**Marital Status**	Single	511	79.7
	Married	120	18.7
	Widowed	1	0.2
	Divorced	9	1.4
**Employment Status**	Yes	242	37.8
	No	399	62.2
**Monthly income**	9999 SAR or less	166	25.9
	10,000–19,999 SAR	95	14.8
	20,000 SAR or more	63	9.8
	Preference not to disclose	130	20.3
	No monthly income	187	29.2
**Educational Level**	High school diploma or less	216	33.7
	Bachelors	330	51.5
	Higher education	95	14.8
**Province**	Riyadh	323	50.4
	Makkah	39	6.1
	Al-Medina	31	4.8
	Al-Baha	4	0.6
	Al-Jouf	4	0.6
	Qassim	30	4.7
	Hail	2	0.3
	Tabouk	4	0.6
	Aseer	12	1.9
	Najran	2	0.3
	Eastern Province	181	28.2
	Northern Borders Province	5	0.8
	Jazan	4	0.6
BMI **	Below 18.5	114	17.8
	18.5–24.9	250	39.0
	25.0–29.9	148	23.1
	30.0–34.9	74	11.5
	35.0–39.9	35	5.5
	Above 40	20	3.1
**Most frequently used social media application**	Snapchat	124	19.3
	TikTok	177	27.6
	Twitter	105	16.4
	Instagram	118	18.4
	YouTube	16	2.5
	Facebook	3	0.5
	LinkedIn	13	2.0
	WhatsApp	68	10.6
	Telegram	9	1.4
	Tumblr	1	0.2
	Reddit	1	0.2
	None	6	0.9

* Abbreviations: BMI, body mass index (kg/m^2^); SAR, Saudi Riyal. ** Notes: BMI categories are based on standard WHO cutoffs.

**Table 2:** Highest endorsed EAT-26 items in the study sample (*n* = 641) *.

Item No.	EAT-26 Item	Subscale	% Often–Always
12	I think about burning calories when I exercise	Dieting	63.2
1	I am terrified about being overweight	Dieting	59.6
14	I am preoccupied with the thought of having fat on my body	Dieting	57.4
11	I am occupied with a desire to be thinner	Dieting	54.9
22	I feel uncomfortable after eating sweets	Dieting	52.4
19	I display self-control around food.	Oral control	52.1
13	Other people think that I am too thin	Oral control	48.5
15	I take longer than others to eat my meals	Oral control	47.9
18	I feel that food controls my life.	Bulimia/Food preoccupation	43.2
20	I feel that others pressure me to eat	Oral control	42.7

* EAT-26 = Eating Attitudes Test-26. Values show the percentage of respondents endorsing each item as ‘Often’, ‘Usually’, or ‘Always’ (collapsed). The complete item-level distribution for all 26 items is provided in Supplementary Table S1.

**Table 3:** Social media engagement across daily routines (SMEQ items) (*n* = 641) *.

Routine	Mean ± SD (Times/Week)	% Every Day	% ≥5 Days/Week
At bedtime	5.0 ± 2.4	52.6	65.1
During breakfast	3.5 ± 2.7	27.5	37.6
During dinner	3.7 ± 2.7	28.5	42.3
Within 15 min of waking	4.0 ± 2.7	34.9	48.7
During lunch	3.4 ± 2.7	27.6	38.5

* SMEQ, Social Media Engagement Questionnaire; SD, standard deviation. Values show mean engagement (times/week) and selected high-frequency indicators. Detailed frequency distributions by routine are provided in Supplementary Table S2.

**Table 4:** Multiple linear regression models predicting EAT-26 total score and subscale scores (*n* = 634) *.

Predictor	B	SE	t	*p*-Value	β	95% CI
**Panel A. EAT-26 Total**						
Gender (male vs. female)	−1.111	1.250	−0.889	0.374	−0.071	[−0.228, 0.086]
BMI	0.340	0.113	2.998	0.003 **	0.125	[0.043, 0.206]
Composite SM score	−0.267	0.058	−4.579	<0.001 **	−0.181	[−0.258, −0.103]
Age group	−2.240	0.962	−2.328	0.020 **	−0.096	[−0.177, −0.015]
Model fit: R^2^ = 0.045; Adjusted R^2^ = 0.039						
**Panel B. Dieting**						
Gender (male vs. female)	−1.16	0.71	−1.64	0.102	−0.13	[−0.28, 0.03]
BMI	0.40	0.06	6.19	<0.001 **	0.25	[0.17, 0.33]
Composite SM score	−0.14	0.03	−4.13	<0.001 **	−0.16	[−0.24, −0.08]
Age group	−0.65	0.55	−1.19	0.236	−0.05	[−0.13, 0.03]
Model fit: R^2^ = 0.080; Adjusted R^2^ = 0.075						
**Panel C. Bulimia/Food Preoccupation**						
Gender (male vs. female)	−0.30	0.34	−0.87	0.384	−0.07	[−0.23, 0.09]
BMI	0.11	0.03	3.58	<0.001 **	0.15	[0.07, 0.23]
Composite SM score	−0.06	0.02	−3.60	<0.001 **	−0.14	[−0.22, −0.06]
Age group	−0.96	0.26	−3.63	<0.001 **	−0.15	[−0.23, −0.07]
Model fit: R^2^ = 0.045; Adjusted R^2^ = 0.039						
**Panel D. Oral Control**						
Gender (male vs. female)	0.35	0.38	0.93	0.355	0.07	[−0.08, 0.23]
BMI	−0.17	0.03	−4.95	<0.001 **	−0.20	[−0.28, −0.12]
Composite SM score	−0.07	0.02	−4.12	<0.001 **	−0.16	[−0.24, −0.08]
Age group	−0.63	0.29	−2.17	0.031 **	−0.09	[−0.17, −0.01]
Model fit: R^2^ = 0.078; Adjusted R^2^ = 0.072						

* 1. B = unstandardized coefficient; β = standardized coefficient; CI = 95% confidence interval. 2. Composite SM score reflects the summed frequency-based SMEQ routine items. 3. Reference category for gender: female (male–female shown). 4. Regression models were estimated using complete-case data (*n* = 634) due to missing values in one or more model variables. ** These values are statistically significant (*p*-value < 0.05).

## Data Availability

The raw data supporting the conclusions of this article will be made available by the authors on request.

## Supplementary Materials

The following supporting information can be downloaded at: https://www.mdpi.com/article/10.3390/healthcare14050666/s1, Table S1: Scores of the Eating Attitudes Test-26 (EAT-26) questionnaire among participants (*n* = 641); Table S2: Scores of the Social Media Engagement Questionnaire (SMEQ) among participants (*n* = 641).
