# Supplementary material for: Social Media and High-Risk Eating Behaviors in Adults: A Cross-Sectional Study
Source: Healthcare (Basel). 2026 Mar 6;14(5):666. doi: 10.3390/healthcare14050666 (PMC12984588; doi:10.3390/healthcare14050666)
Supplement: Supplementary file 1 [file healthcare-14-00666-s001.zip › healthcare-4092817-supplementary.pdf]

Supplementary Materials

Supplementary Table S1: Scores of the Eating Attitudes Test-26 (EAT-26) questionnaire among participants (N = 641).

No.	EAT-26 Items	Subscale	Never		Rarely		Sometimes		Often		Usually		Always		Mean ± std
			N	%	N	%	N	%	N	%	N	%	N	%	
1	**I am terrified about being overweight**	Dieting	106	16.5%	57	8.9%	96	15.0%	111	17.3%	116	18.1%	155	24.2%	2.84 ±1.767
2	**I avoid eating when I am hungry**	Oral Control	214	33.4%	149	23.2%	118	18.4%	79	12.3%	60	9.4%	21	3.3%	1.51 ±1.457
3	**I find myself preoccupied with food**	Bulimia and food preoccupation	88	13.7%	111	17.3%	173	27.0%	112	17.5%	83	12.9%	74	11.5%	2.33 ±1.532
4	**I have gone on eating binges where I feel that I may not be able to stop**	Bulimia and food preoccupation	173	27.0%	142	22.2%	131	20.4%	76	11.9%	70	10.9%	49	7.6%	1.8 ±1.584
5	**I cut my food into small pieces.**	Oral Control	161	25.1%	95	14.8%	134	20.9%	101	15.8%	85	13.3%	65	10.1%	2.08 ±1.659
6	**I am aware of the calorie content of foods that I eat**	Dieting	210	32.8%	89	13.9%	101	15.8%	112	17.5%	72	11.2%	57	8.9%	1.87 ±1.692
7	**I particularly avoid food with a high carbohydrate**	Dieting	242	37.8%	90	14.0%	111	17.3%	101	15.8%	49	7.6%	48	7.5%	1.64 ±1.632

	**content (i.e. bread, rice, potatoes, etc.)**													
8	**I feel that others would prefer if I ate more.**	Oral Control	219	34.2 %	68	10.6 %	96	15. 0%	105	16. 4%	83	12.9 %	70	10.9 %	1.96 ±1.772
9	**I vomit after I have eaten**	Bulimia and food preoccupati on	425	66.3 %	39	6.1%	55	8.6 %	68	10. 6%	36	5.6%	18	2.8%	0.92±1.457
10	**I feel extremely guilty after eating**	Dieting	212	33.1 %	92	14.4 %	80	12. 5%	102	15. 9%	65	10.1 %	90	14.0 %	1.98±1.816
11	**I am occupied with a desire to be thinner**	Dieting	158	24.6 %	59	9.2%	72	11. 2%	96	15. 0%	83	12.9 %	173	27.0 %	2.63±1.944
12	**I think about burning up calories when I exercise**	Dieting	120	18.7 %	45	7.0%	71	11. 1%	101	15. 8%	120	18.7 %	184	28.7 %	2.95±1.848
13	**Other people think that I am too thin**	Oral Control	152	23.7 %	74	11.5 %	104	16. 2%	106	16. 5%	94	14.7 %	111	17.3 %	2.39±1.797
14	**I am preoccupied with the thought of having fat on my body**	Dieting	117	18.3 %	73	11.4 %	83	12. 9%	92	14. 4%	108	16.8 %	168	26.2 %	2.79±1.845
15	**I take longer than others to eat my meals**	Oral Control	132	20.6 %	97	15.1 %	105	16. 4%	114	17. 8%	89	13.9 %	104	16.2 %	2.38±1.741
16	**I avoid foods with sugar**	Dieting	186	29.0 %	90	14.0 %	120	18. 7%	116	18. 1%	73	11.4 %	56	8.7%	1.95±1.651
17	**I eat diet foods**	Dieting	217	33.9 %	94	14.7 %	118	18. 4%	106	16. 5%	72	11.2 %	34	5.3%	1.73±1.588
18	**I feel that food controls my life.**	Bulimia and food	196	30.6 %	76	11.9 %	92	14. 4%	123	19. 2%	74	11.5 %	80	12.5 %	2.07±1.766

		preoccupation													
19	**I display self-control around food.**	Oral Control	79	12.3%	90	14.0%	138	21.5%	150	23.4%	104	16.2%	80	12.5%	2.55±1.531
20	**I feel that others pressure me to eat**	Oral Control	192	30.0%	87	13.6%	88	13.70%	113	17.6%	98	15.3%	63	9.8%	2.04±1.735
21	**I give too much time and thought to food**	Bulimia and food preoccupation	140	21.8%	113	17.6%	117	18.30%	117	18.30%	86	13.40%	68	10.60%	2.16±1.644
22	**I feel uncomfortable after eating sweets**	Dieting	132	20.6%	100	15.6%	73	11.40%	129	20.10%	90	14.00%	117	18.30%	2.46±1.782
23	**I engage in dieting behavior.**	Dieting	205	32.0%	100	15.6%	98	15.30%	115	17.90%	63	9.80%	60	9.40%	1.86±1.684
24	**I like my stomach to be empty.**	Dieting	169	26.4%	87	13.6%	146	22.80%	113	17.60%	73	11.40%	53	8.30%	1.99±1.603
25	**I have the impulse to vomit after meals.**	Bulimia and food preoccupation	396	61.8%	46	7.20%	50	7.80%	80	12.50%	37	5.80%	32	5.00%	1.08±1.584
26	**I enjoy trying new rich foods**	Dieting	63	9.80%	61	9.50%	116	18.10%	120	18.70%	130	20.30%	151	23.60%	1.99±1.611

Abbreviations: EAT-26, Eating Attitudes Test–26; SD, standard deviation. Notes: Values are presented as N (%). “Mean ± SD” reflects the average raw item response on the 6-point response scale (Never–Always) and is provided for descriptive purposes.

Supplementary Table S2. *Scores of the Social Media Engagement Questionnaire (SMEQ) among participants (N = 641).*

	Not one day N(%)	One day N(%)	Two days N(%)	Three days N(%)	Four days N(%)	Five days N(%)	Six days	Every day N(%)	Mean ± SD
**How many times do you used social media 15 minutes before going to bed?**	42 (6.6)	42 (6.6)	47 (7.3)	66 (10.3)	27 (4.2)	44 (6.9)	36 (5.6)	337 (52.6)	5.0±2.4
**How many times do you use social media during breakfast in the morning?**	122 (19)	80 (12.5)	86 (13.4)	73 (11.4)	39 (6.1)	37 (5.8)	28 (4.4)	176 (27.5)	3.5±2.7
**How many times do you use social media while eating dinner at night?**	116 (18.1)	74 (11.5)	68 (10.6)	69 (10.8)	43 (6.7)	50 (7.8)	38 (5.9)	183 (28.5)	3.7±2.7
**How many times do you use social media within 15 minutes of waking up in the morning?**	76 (11.9)	87 (13.6)	80 (12.5)	63 (9.8)	23 (3.6)	47 (7.3)	41 (6.4)	224 (34.9)	4.0±2.7
**How many times do you use social media while eating lunch?**	138 (21.5)	70 (10.9)	78 (12.2)	75 (11.7)	33 (5.1)	40 (6.2)	30 (4.7)	177 (27.6)	3.4±2.7

Abbreviations: SMEQ, Social Media Engagement Questionnaire; SD, standard deviation. Notes: Response options reflect frequency of use in days per week (0 = not one day to 7 = every day). Values are presented as N (%). "Mean ± SD" reflects the mean number of days/week for each routine item.
